# Biomass Alginate Derived Oxygen-Enriched Carbonaceous Materials with Partially Graphitic Nanolayers for High Performance Anodes in Lithium-Ion Batteries

**DOI:** 10.3390/nano13010082

**Published:** 2022-12-24

**Authors:** Xiaolei Sun, Yao Chen, Yang Li, Feng Luo

**Affiliations:** 1 Tianjin Key Lab for Rare Earth Materials and Applications, Center for Rare Earth and Inorganic Functional Materials, School of Materials Science and Engineering, Nankai University, Tianjin 300350, China; 2 The State Key Laboratory of Refractories and Metallurgy, College of Materials and Metallurgy, Wuhan University of Science and Technology, Wuhan 430081, China; 3 Institute for Integrative Nanosciences, Leibniz Institute for Solid State and Materials Research Dresden, 01069 Dresden, Germany

**Keywords:** oxygen-enriched carbon, hierarchically porous structure, lithium storage, long cycle life, high-rate capability

## Abstract

Lithium-ion batteries with high reversible capacity, high-rate capability, and extended cycle life are vital for future consumer electronics and renewable energy storage. There is a great deal of interest in developing novel types of carbonaceous materials to boost lithium storage properties due to the inadequate properties of conventional graphite anodes. In this study, we describe a facile and low-cost approach for the synthesis of oxygen-doped hierarchically porous carbons with partially graphitic nanolayers (Alg-C) from pyrolyzed Na-alginate biopolymers without resorting to any kind of activation step. The obtained Alg-C samples were analyzed using various techniques, such as X-ray diffraction, Raman, X-ray photoelectron spectroscopy, scanning electron microscope, and transmission electron microscope, to determine their structure and morphology. When serving as lithium storage anodes, the as-prepared Alg-C electrodes have outstanding electrochemical features, such as a high-rate capability (120 mAh g^−1^ at 3000 mA g^−1^) and extended cycling lifetimes over 5000 cycles. The post-cycle morphologies ultimately provide evidence of the distinct structural characteristics of the Alg-C electrodes. These preliminary findings suggest that alginate-derived carbonaceous materials may have intensive potential for next-generation energy storage and other related applications.

## 1. Introduction

After finding widespread usage in a broad range of portable electronic gadgets and power tools, rechargeable lithium-ion batteries, often known as LIBs, will eventually find key uses in larger-scale battery storage and electric vehicles [1,2,3]. In this regard, they outperform other rechargeable battery types, including lead–acid, nickel–cadmium nickel–metal hydride batteries, and so on [4,5]. Graphite is now the most preferred choice for anode materials in LIBs due to its outstanding properties, which include a relatively low operating voltage, cycle stability, and minimal capacity loss for storing electrical charge [6,7]. Despite this, there are still a few drawbacks to it. Among these problems are an inferior rate capability and a low theoretical specific capacity, which comes in at around 372 mAh g^−1^ [8]. In addition, graphite has the potential to create issues with lithium dendrites since it is a conductive material. Because of these unfavorable qualities, graphite is unable to fulfill the enormous demand for next-generation LIBs in the rapidly expanding market for consumer devices [9,10,11]. As a result, other new forms of carbonaceous materials with varying textures and morphologies, such as porous carbon materials, active carbon, carbon nanotubes, carbon nanofibers, graphene, and hybrid carbon, have been developed as excellent electrode materials for the purpose of improving lithium storage capacity [12,13,14,15,16,17]. Porous carbon nanomaterials stand out among all these potential anode materials because of the fascinating properties they possess, including high electrical conductivity, well-developed microstructure, and excellent chemical stability [17,18,19]. This attention has been garnered over the course of the past few decades. The electrochemical characteristics of carbonaceous electrodes have been the subject of significant research, which has led to the development of a number of different approaches [20,21,22]. Improving carbon-based electrodes by designing hierarchical three-dimensional (3D) porous nanostructures is a beneficial method [17,23]. These nanostructures not only offer a continuous electron channel but also improve ion transport by shortening the diffusion paths of electrolytes. Introducing heteroatoms such as nitrogen, phosphorus, and oxygen-rich functional groups (carboxyl, ether, phenol, and so on) into the carbon skeleton is an additional approach [24,25,26]. This strategy focuses on the introduction of heteroatoms. It is believed that the incorporation of heteroatoms or functional groups may increase the wettability of the electrolyte and result in improved extra faradaic reactions [13,18,27].

The aforementioned methods increase electrochemical performance significantly, but they are not without their downsides. Some of these techniques, for instance, might be costly and difficult to scale up because they need unique equipment or intricate manufacturing procedures. Therefore, these difficulties limit their potential real-world applications in the realm of energy storage and conversion. That is why it is more appealing than ever to employ functional group-rich materials as carbon precursors. Instead, biomass resources are thought to adequately cover the needs since they are renewable, low-cost, and ecologically beneficial [28,29,30]. Biomass is an abundant carbon source that may be processed into carbon compounds by carbonization, pyrolysis, and activation techniques. A complex and efficient hierarchical structure with naturally interconnected scaffolds is common in carbon collected from natural resources [31]. The anode materials for lithium storage to date have included a variety of carbon compounds derived from biomass carbon precursors [32,33,34,35]. The biomass-derived carbon materials that have been described thus far, however, tend to have a low capacity and/or an unsatisfactory cycling performance [36,37,38].

Sodium alginate (Alg) is a biomass polysaccharide product extracted from brown seaweed. From a chemical standpoint, Na alginate is mostly composed of sodium salt alginate, which is a copolymer comprising β-D-mannuronic acid and α-L-guluronic acid that may give oxygen-rich functional groups [39,40]. Inspired by this kind of biomaterial, recent studies have shown that oxygen-rich carbon can be fabricated by carbonizing solid Na-containing organic compounds at temperatures above 600 °C and applied to supercapacitors [41,42,43]. However, there are relatively few examples of the use of Na-alginate to prepare carbon-based anode materials that have a high capacity for storing lithium ions. In this work, we present a facile approach for the synthesis of oxygen-doped hierarchically porous carbons with partially graphitic nanolayers (Alg-C) from a Na-alginate biopolymer by a pyrolysis process. This method does not require the assistance of any additional activation process, which simplifies the procedure and decreases the cost. When tested as an anode material for lithium storage, the Alg-C electrode demonstrates exceptionally good electrochemical properties such as high-rate discharge/charge performance (120 mAh g^−1^ at 3000 mA g^−1^) and long cycling lifetimes of 5000 cycles. Thus, this suggests that the Alg-C material has tremendous promise in the future application of high-rate LIBs.

## 2. Materials and Methods

### 2.1. Materials Preparation

The Alg-C product was synthesized by directly carbonizing Na-alginate powder. A particular quantity of raw material was placed in a quartz boat and heated in a horizontal tube furnace during a typical procedure. Under an argon environment (200 mL min^−1^), the pyrolyzed process was maintained at 550 °C at a heating rate of 10 °C min^−1^ for 2 h. After naturally cooling to ambient temperature, the powder was treated many times with deionized water to eliminate inorganic impurities such as sodium salt Na_2_CO_3_. The dark carbon was then dried in an oven at 80 °C for 12 h and denoted as Alg-C.

### 2.2. Materials Characterization

X-ray powder diffraction (XRD) patterns of the as-prepared products were collected on a Rigaku Smart Lab Diffractometer with a filtered Cu Kα (λ = 1.5406 Å) radiation source operating at a tube voltage of 40 kV and current of 40 mA. The Raman spectra were recorded by a TEO SR-500I-A spectrometer using an excitation source of 532 nm and 50 mW laser power. Silicon was utilized as a reference for calibration. X-ray photoelectron spectroscopy (XPS) data were explored further using an ESCALAB 250Xi spectrometer (Thermo Scientific, Waltham, MA, USA) equipped with an Al Kα monochromatic source (1486.7 eV). The morphology and microstructure were identified using a JSM-7800 field-emission scanning electron microscope (SEM, 15 kV, JEOL Ltd., Tokyo, Japan) connected with an energy dispersive X-ray analyzer and a JEM-2800 transmission electron microscope (TEM, 200 kV, JEOL Ltd., Tokyo, Japan). The nitrogen adsorption/desorption isotherm data were obtained using an ASAP 2020 Micromeritics instrument at -196.15 °C. On a TG-DTA 8122 instrument (Rigaku, Tokyo, Japan), thermogravimetric analysis (TGA) measurement was conducted at a constant heating rate of 10 °C min^−1^ at a flow rate of 100 mL min^−1^ in a nitrogen environment. At room temperature, Fourier transform infrared (FTIR) spectroscopy was performed using an FTIR spectrometer (Nicolet iS50, Thermo Scientific, Waltham, MA, USA) with wavenumbers between 500 and 4000 cm^−1^.

### 2.3. Electrochemical Measurements

Electrochemical studies were conducted using Swagelok-type cells at room temperature. Active materials (80 wt%), acetylene black (AB, 10 wt%), and polyvinylidene fluoride (PVDF, 10 wt%) binder were added to an N-methylpyrrolidone (NMP) solvent to make a slurry. The resulting slurry was then cast evenly using a doctor blade onto a copper foil substrate, dried under an infrared lamp to remove residual solvents, and then dried at 120 °C under vacuum for 12 h. Whatman GF/D porous glass filter paper was used directly as the separator, whereas lithium ribbon (99.9% trace metals basis, China Energy Lithium Co., Ltd., Tianjin, China) acted as both counter/reference electrodes. The electrolyte was a mixture of ethylene carbonate (EC), ethyl methyl carbonate (EMC), and dimethyl carbonate (DMC) with one molar LiPF_6_ (1:1:1 by volume). All half-cells with prepared Alg-C electrodes were constructed in a glove box (MIKROUNA, Shanghai, China) under argon with oxygen and moisture levels of less than 0.1 ppm. Note that 100 μL of electrolyte was consistently supplied to each half-cell using a microsyringe. Commercial graphite and multi-wall carbon nanotubes (MWCNTs) from Aladdin Ltd. (Shanghai, China) were also evaluated in this study under the same circumstances for comparison. The loading amount of active materials for all electrodes was 1.0 ± 0.1 mg cm^−2^. Prior to conducting electrochemical tests, all manufactured cells were aged for 12 h to guarantee adequate electrolyte solution soaking. Using a multichannel battery tester (CT3002A, LANHE, Wuhan, China), galvanostatic discharge/charge studies were conducted with current densities ranging from 100 to 3000 mA g^−1^ in the voltage window of 0.02 to 3.0 V (against Li/Li^+^). Cyclic voltammetry (CV) investigations were performed at different sweep speeds ranging from 0.1 to 200 mV s^−1^ within a cut-off voltage window of 0.02 to 2.0 V. Electrochemical impedance spectroscopy (EIS) was performed at the open circuit potential using a 5.0 mV sine wave between 100 kHz and 10 mHz. CV and EIS measurements were performed using an electrochemical workstation (CHI660E, CHI Instruments, Shanghai, China) at room temperature. To analyze the lithium insertion/deinsertion into Alg-C anodes, the charged cells were physically opened in a glove box under an argon environment for different cycle counts, followed by multiple washes of the cycling electrode with DMC to eliminate residues, and dried for preservation. Then, using SEM and TEM analysis, the post morphologies of the cycled anodes were examined further.

## 3. Results and Discussion

**Figure 1:**
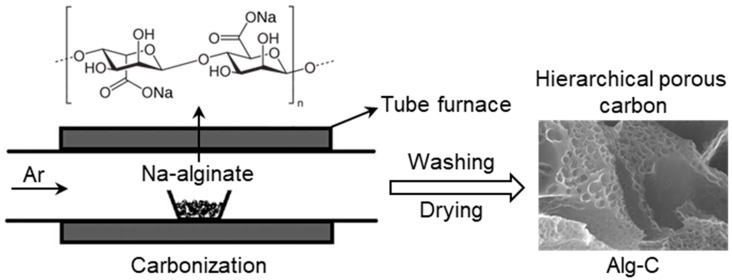
Schematic illustration of the preparation process of carbonaceous Alg-C material.

Figure 1 is a schematic depiction of the hierarchical porous carbon preparation process. The carbonaceous Alg-C material was produced by carrying out a straightforward carbonization procedure on a Na-alginate biopolymer in a tube furnace at 550 °C for 2 h while nitrogen was present in the atmosphere. As the temperature continues to rise, Na-alginate begins to carbonize, resulting in the release of water, carbon dioxide, and carbon monoxide, which kinetically promotes the formation of porous structures. In addition, Na_2_CO_3_ will form and be held in place within the carbon framework. The last step, which is to remove any remaining inorganic impurities such as sodium salt Na_2_CO_3_ by washing the black material with deionized water, ultimately results in the formation of porous carbonaceous Alg-C material.

**Figure 2:**
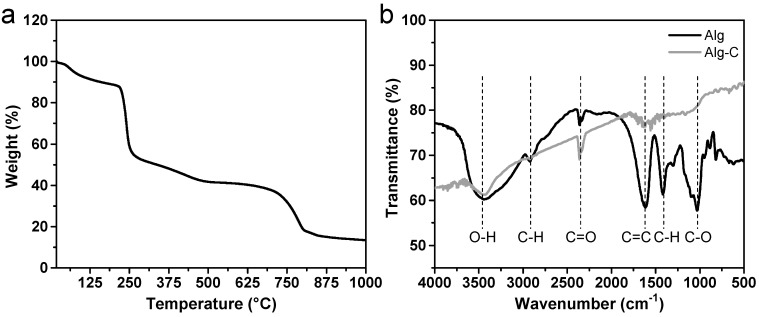
(**a**) TGA curve of Na-alginate biopolymer obtained from brown algae. (**b**) FTIR spectra of Na-alginate and its derived carbonaceous Alg-C material.

The TGA methodology is a way of assessing the change in mass as a result of an increase in temperature. It is an essential instrument for comprehending the intrinsic characteristics of carbon-based compounds, in addition to the purity of these materials. In the process of converting biomass to carbon via a process known as carbonization, it is conceivable, depending on the nature of the biomass, that some metals or metal ions as well as carbonaceous contaminants will be present. The TGA curve can be seen in Figure 2a. The pyrolysis of Na-alginate has been the subject of substantial research in earlier work and may be broken down into three primary steps (50–200 °C, 200–250 °C, 250–800 °C) [44]. On the TGA curve at temperatures up to 200 °C, a modest weight loss of around 11.5% in the first step corresponds to the removal of water that is only weakly adsorbed on the product surface. The breaking of glycosidic bonds and the production of stable intermediates are both responsible for the considerable mass loss that occurs during the second step, which takes place at temperatures between 200 and 250 °C. The intermediates are subjected to oxidation and carbonization in the third step, indicating the formation of sodium oxide and tiny carbon molecules. Concurrently, the redox reaction that takes place between carbon and sodium oxide results in the development of microporous structures. After 800 °C, a mass loss occurs due to the breakdown of sodium carbonate into carbon dioxide and sodium oxide as well as the sublimation of tiny molecules of carbon [43,44].

FTIR measurements provide further evidence that distinct functional groups are present both before (in the raw material) and after (in Alg-C) the carbonization process. Figure 2b demonstrates that the O-H stretching that is detected at a frequency of around 3446 cm^−1^ in both of the samples could be mostly due to hydroxyl groups and chemisorbed water molecules on carbon. For the raw Na-alginate sample, the FTIR spectrum (black line) reveals many peaks at 3446, 2925, 2349, 1613, 1412, and 1030 cm^−1^. These peaks are caused by a variety of oxygen-related groups, including -OH, -CH, C=O, C=C, C-H, and C-O [45]. When compared to pure Na-alginate, the produced Alg-C sample (gray line) prepared by high-temperature carbonization reveals a small amount of oxygen-related groups with lower intensities. This clearly shows that the amount of oxygen-containing functional groups on the Alg-C carbon surface is dramatically reduced throughout the pyrolysis process.

**Figure 3:**
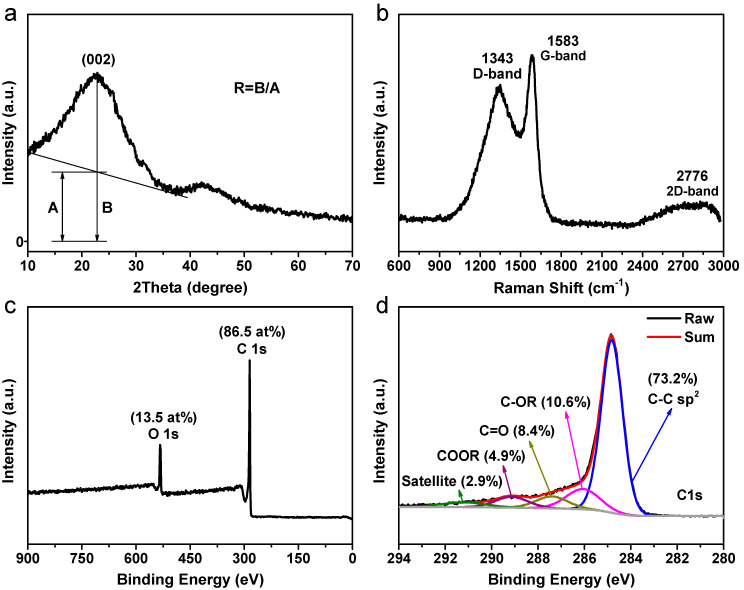
Structure and composition analysis of alginate-derived Alg-C carbon. (**a**) XRD pattern; (**b**) Raman spectrum, XPS full spectrum (**c**), and (**d**) high-resolution of C 1s spectrum.

The XRD pattern in Figure 3a reveals a substantially broadened (002) reflection of graphite at approximately 22.7°, which is characteristic of nongraphitic carbon. Another peak can be seen at about 42.3°, indicating that the material in question is made up of smaller domains of organized graphitic sheets. The experimental (002) peak-to-background ratio, also known as the *R* parameter, is used to determine the number of ordered graphitic sheets. As a result, this value is calculated to be ~2.4, which is smaller than the values reported in earlier research [46]. According to previous studies, porous carbons with lower *R* values are thought to be more effective in LIBs [12,47]. It is thus to be anticipated that the presence of this carbonaceous material would result in noticeable changes to the electrochemical behaviors. 

As a powerful tool for investigating the structures of carbon-containing products, Raman spectroscopy is frequently used. The Raman spectrum of the sample taken at room temperature is shown in Figure 3b for the purpose of performing more research on the product. Two peaks can be distinguished and are located at about 1343 and 1583 cm^−1^. The former band, referred to as the D-band, is located at 1343 cm^−1^, and it is connected to the breathing modes of sp^2^ atoms in rings. This is directly connected to defects or disorders. The latter band, known as the G-band and centered at 1583 cm^−1^, is thought to be caused by the stretching of symmetric bonds among all the sp^2^ carbon atoms [12]. As a result of the decreased carbonization temperature, the D-band and the G-band are much wider than those described in previous research. In general, the integrated intensity (area) ratio between the D- and G-bands (*A_D_/A_G_*) offers a helpful metric for evaluating the degree of the crystalline structure of carbonaceous materials [48]. According to the calculations, the sample has an *A_D_/A_G_* area ratio of ~2.88, which indicates the creation of short-range graphitic carbon with a great deal of structural defects [18]. There was some speculation that this may be linked to the disordered structure with a high doping level of oxygen atoms that was created by self-chemical activation driven by intrinsic alkali. This activation was thought to have come from the one-of-a-kind composition of raw Na-alginate. Furthermore, a broad and weak 2D-band located at around 2776 cm^−1^ was observed, confirming the formation of a layered graphitic-like structure [49]. The findings of the XRD experiment are in reasonable accord with the observations.

In addition, XPS was utilized to further analyze the chemical compositions as well as the states of the sample. Figure 3c shows the XPS survey spectrum obtained from the Alg-C sample. This spectrum reveals that the only elements present on the surface are carbon and oxygen. There is still a significant quantity of oxygen (13.5 at%) present in the carbon structure. Figure 3d shows that the high-resolution C 1s peak can be divided into five fitting subpeaks. It is possible to deduce from the plots that in addition to the majority of the C-C sp^2^ bond (284.8 eV, 73.2 at%), other chemical states that bond to oxygen are also present. These states include C-OR (~286.1 eV, 10.6 at%), C=O (~287.4 eV, 8.4 at%), COOR (~289.1 eV, 4.9 at%), and a satellite peak (~291.2 eV, 2.9 at%). The reversible pseudocapacitive processes are significantly aided by the participation of these oxygen-containing functional groups [50].

**Figure 4:**
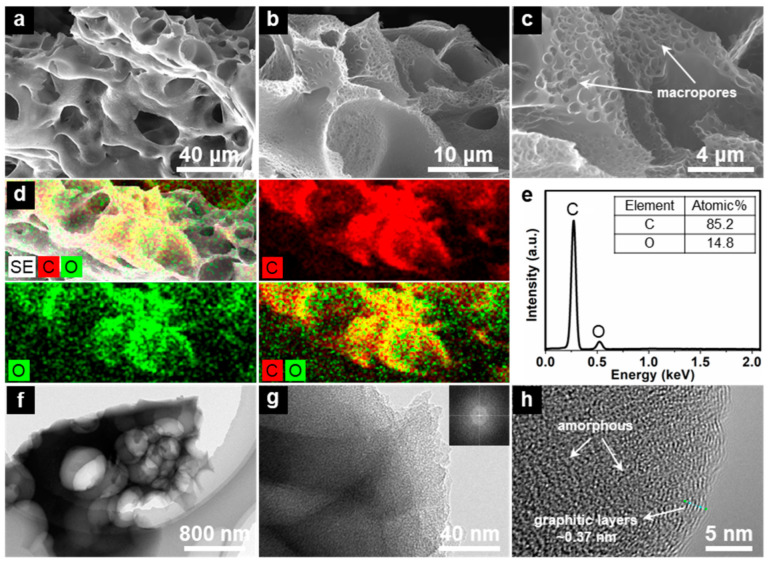
Representative morphological characterizations of Alg-C material. (**a**–**c**) SEM images under different magnifications. (**d**) SEM-EDX micrograph and corresponding elemental mapping images of carbon and oxygen and (**e**) EDX spectrum. (**f**–**h**) TEM images recorded at different magnifications. Inset: FFT of the image of (**g**).

Figure 4 shows the SEM and TEM images of the as-fabricated Alg-C product. It would seem that the Alg-C sample has a 3D structure that is porous and interconnected. A number of macropores that are capable of functioning as electrolyte reservoirs are present in the 3D structure of Alg-C (Figure 4a–c). These macropores should have been created as a result of the dissolving of in situ formed Na_2_CO_3_ particles that took place during the H_2_O wash procedure. As seen in Figure 4d, the energy dispersive X-ray (EDX) elemental mapping results of Alg-C reveal that elemental carbon and oxygen are distributed evenly throughout the material. In addition, the comparable EDX pattern that can be seen in Figure 4e provides further and compelling evidence for the existence of carbon and oxygen components. The calculated percentages of each ingredient are shown in the inset of Figure 4e. According to the results of the semiquantitative examination, the as-synthesized Alg-C material has about 85.2 at% carbon and 14.8 at% oxygen, which is consistent with the abovementioned XPS surface analysis (Figure 3c).

In addition, the TEM images shown in Figure 4f,g illustrate that the sample consists of multiple macropores with thin walls. These macropores have the potential to expose more easily accessible active sites and enhance the transit of electrolyte molecules. The pattern of the fast Fourier transform (FFT), which can be seen in the upper-right inset of Figure 4g, demonstrates the existence of an apparent amorphous phase in the Alg-C product. Figure 4h is a high-resolution TEM image that reveals many micropores in the Alg-C sample. It also reveals that the structure of the amorphous carbon material contains graphitic nanolayers in part. The graphitic carbon that was produced from doped-heteroatoms and the production of defects is scattered near the edge of the pore structure, while the disordered carbon is positioned away from the pore structure. The measured interplanar distance is 0.37 nm, which corresponds to the spacing of the (002) planes, which is greater than that of graphite (*d*_002_ = 0.34 nm). It is well known that Na_2_CO_3_ has a strong effect on the graphitization of carbon [51]. Therefore, the carbon atoms produced during the carbonization of Na-alginate will grow into short-rang graphitic layers under the catalysis of the in situ formed Na_2_CO_3_ nanoparticles. In a structure that combines graphitic and disordered carbon, graphitic carbon contributes superior electrical conductivity and stability, while disordered carbon provides an abundance of active sites due to its large surface area [23,52].

**Figure 5:**
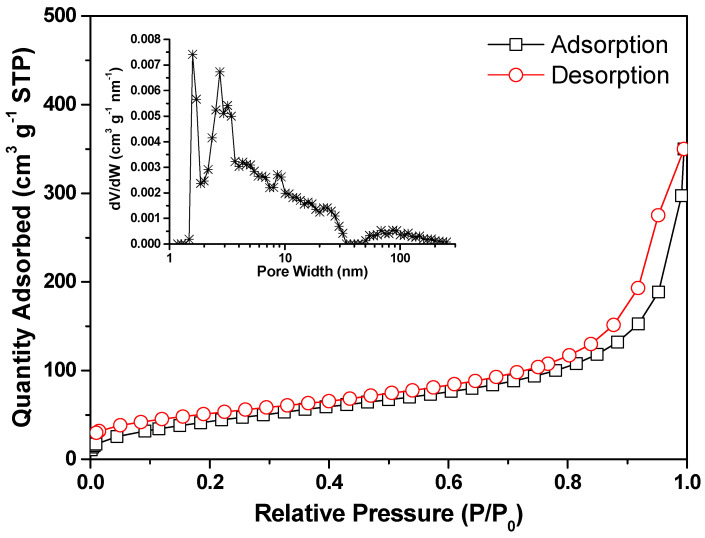
Nitrogen adsorption/desorption isotherms and the corresponding pore-size distribution (inset) of the Alg-C material.

As predicted, the formation of such a unique structure results in a significant increase in the Brunauer–Emmett–Teller (BET) specific surface area, which is approximately 226.8 m^2^ g^−1^ (Figure 5). This value is somewhat lower than the carbon materials that are reported to be generated at higher activation temperatures [41]. The inset pore size distribution estimated using the Barrett–Joyner–Halenda (BJH) method confirms the co-existence of nanopores of 1–10 nm in size and large pores over 100 nm. A large surface area and well-developed macropores and micropores may enhance electrolyte ion diffusion to active areas with decreased ion-transport resistance and may provide more active sites to enhance energy storage performance [53,54]. In addition, the partially graphitic nanolayers with broad interlayer distances might provide a reliable pathway for charge carrier transport, which enables the fast discharge/charge capabilities.

**Figure 6:**
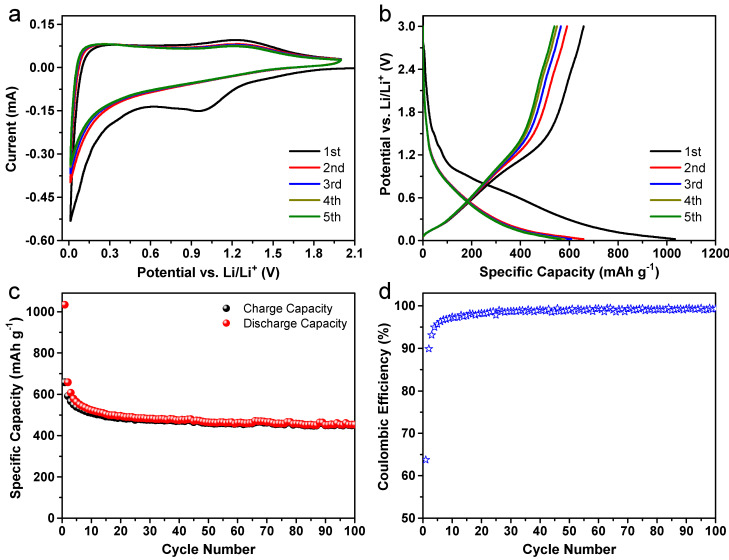
Electrochemical performance of Alg-C electrodes in Li half-cells. (**a**) The initial five-cycle CV profiles within the potential range of 0.02–2.0 V at a scan rate of 0.2 mV s^−1^. (**b**) Galvanostatic discharge/charge curves for the first five cycles. (**c**) Cycling performance at a current density of 100 mA g^−1^ with its (**d**) Coulombic efficiency in the potential window of 0.02–3.0 V.

Figure 6a depicts the first five consecutive CV cycles that were performed at a scan rate of 0.2 mV s^−1^ in the potential window of 0.02–2.0 V vs. Li/Li^+^. The CV profiles of the Alg-C electrode are representative of those seen in carbonaceous anode materials in general. When compared to the following four cycles, the first cycle exhibits two extra cathodic current peaks, centered at roughly 1.41 and 0.99 V, respectively. These peaks do not appear in the subsequent cycles, which could be related to the breakdown of the linear carbonate solvents that are present in the EMC and DMC of the electrolyte [12]. Furthermore, the decrease in the current below 1.5 V in the first cycle is significantly greater than the values in the subsequent four cycles. This additional current in the first cycle can be attributed to the irreversible reduction of the electrolyte at low voltage to form a protective solid electrolyte interphase (SEI) layer [18]. The partial breakdown of SEI components such as LiF, Li_2_CO_3_, and RCO_2_Li is connected to the presence of a wide oxidation peak at a potential of less than 1.3 V in the anodic processes that are present in the following curves [5,18]. After the first cycle, it is clear that the subsequent CV curves are relatively comparable to one another, which indicates that both the formed SEI layer and the Alg-C electrode are very stable.

Figure 6b depicts the galvanostatic discharge/charge profiles of the Alg-C electrode at a current density of 100 mA g^−1^ for the first five cycles within a potential range of 0.01 to 3.0 V. As seen, the initial discharge specific capacity can reach 1033 mAh g^−1^ in the first cycle. This value was determined by using the following formula: *C* = *Q*/*m*, where *Q* is the actual capacity in mAh, and *m* is the weight of the active material in g [55]. During the subsequent charge process, it is still capable of maintaining a high reversible specific capacity of around 659 mAh g^−1^. This value is nearly 1.8 times greater than that of representative graphite (LiC_6_, 372 mAh g^−1^). This results in an initial Coulombic efficiency of 63.8% due to the irreversible capacity. The ratio of the quantity of useable charge to the amount of stored charge is the definition of Coulombic efficiency [56]. Another way to express this concept is to say that the charge capacity is divided simply by the discharge capacity in the half cell. In fact, the significant and permanent loss of capacity is something that is to be anticipated for carbonaceous anodes used in LIBs [23,52]. The poor efficiency value is usually thought to be caused by interfacial processes, as well as the irreversible insertion of lithium ions into certain undetermined places in Alg-C during the initial discharge phase. This is in accordance with the general consensus among researchers [28,32,36]. The Coulombic efficiency of carbonaceous-based electrodes is a significant factor for practical applications. In addition, to fulfill the demands of the commercial market for LIBs, further research should be focused on looking at how to improve the initial Coulombic efficiency *via* prelithiation and/or some further adjustments [18]. The subsequent cycling profiles suggest that the lithiation and delithiation processes are operating at a steady level. Even after the fifth cycle, the charge specific capacity can be maintained at 539 mAh g^−1^ with a high Coulombic efficiency of about 95.6%. In addition, these parallel characteristics are in accordance with the findings that are gleaned from the CV curves. Figure 6c shows that the Alg-C electrode has excellent cycling performance and reversibility when subjected to long cycles. A specific high reversible specific capacity of 454 mAh g^−1^ is achieved even after 100 cycles. This indicates that the anodic performance is much superior to that of graphite anodes. In addition, the Coulombic efficiency increases rapidly up to 98.7% after 30 cycles and then stabilizes at around 99.2% beyond this point (Figure 6d). This finding is indicative of the ease with which lithium ions may be inserted and extracted, as well as the excellent efficiency of transporting electrons and ions throughout the whole electrode.

**Figure 7:**
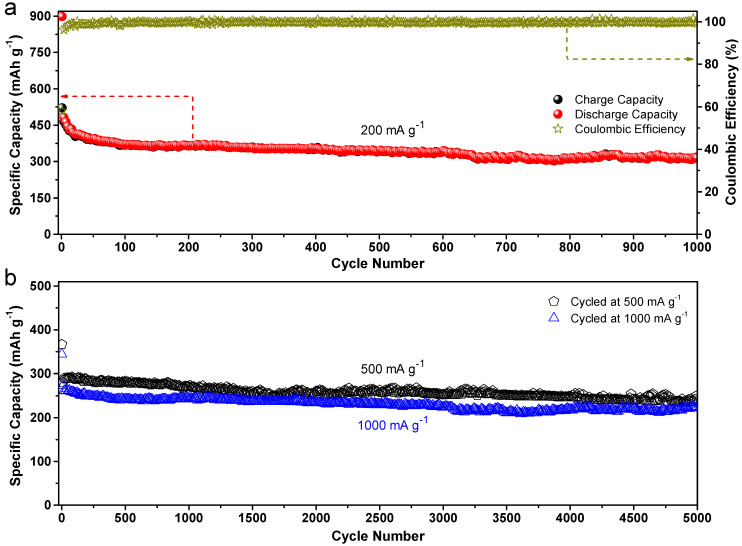
Long cycling performance of Alg-C electrodes. (**a**) At a current density of 200 mA g^−1^ for 1000 cycles and the corresponding Coulombic efficiency. (**b**) At high current densities of 500 and 1000 mA g^−1^ for 5000 cycles after aging for three cycles at 100 mA g^−1^.

Galvanostatic discharge/charge measurements were performed at various high current densities between 0.02 and 3.0 V to provide further evidence that supports the great endurance of the Alg-C electrode. The performance of the battery during long-term cycling is depicted in Figure 7a up to the 1000th cycle when measured at a current density of 200 mA g^−1^. After 1000 cycles, the charge specific capacity is still 315 mAh g^−1^, which corresponds to a capacity retention of 60.5% and a small specific capacity loss of 0.2 mAh g^−1^ every cycle. In addition, the Coulombic efficiency is maintained at a level higher than 99.6% after the first few cycles, which coincides with the obtained reliable cycling performance.

The Alg-C electrode cyclability was further tested upon 5000 cycles, as depicted in Figure 7b. This was done to further validate the longevity of this Alg-C electrode to function at higher current densities (500 and 1000 mA g^−1^). After activating both cells for three cycles at a small current density of 100 mA g^−1^, the experiment continued with either 4997 cycles at 500 or 1000 mA g^−1^. It is clearly seen that the initial reversible specific capacities at 500 and 1000 mA g^−1^ are 367 and 344 mAh g^−1^, respectively, with specific capacity retentions down to 260 and 234 mAh g^−1^ after 2000 cycles, and 238 and 223 mAh g^−1^ after 5000 cycles. The results further demonstrate the excellent cycling stability of Alg-C at different high discharge/charge current densities. Based on these findings, one may conclude that the usage of Alg-C materials as anode electrodes in LIBs has a great deal of potential.

**Figure 8:**
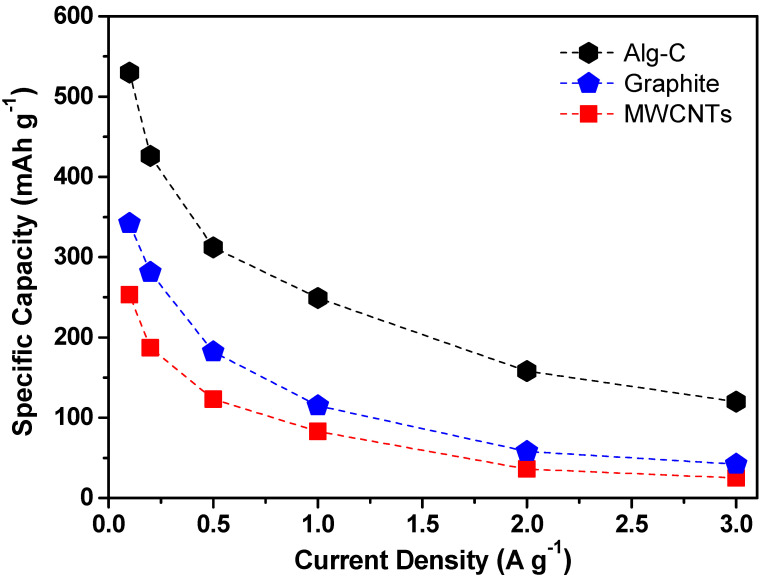
Comparison of the rate capabilities of Alg-C, graphite, and MWCNTs under various current densities within the potential window from 0.02 to 3.0 V.

The rate capability of the Alg-C electrode is yet another remarkable quality of this electrode. As plotted in Figure 8, the capacity of the as-prepared Alg-C is much greater than that of commercial graphite and multi-wall carbon nanotubes (MWCNTs) at all measured current densities from 100 to 3000 mA g^−1^. The reversible specific capacity for Alg-C at a low current density of 100 mA g^−1^ is around 530 mAh g^−1^, which is much larger than that observed for graphite (~342 mAh g^−1^) and MWCNTs (~253 mAh g^−1^). It is important to emphasize that the Alg-C product is partially graphitized, and that the locally graphitic nanolayers are layered in a way that forms turbostratic disorders, as exhibited by Raman spectroscopy and high-resolution TEM results. Generally speaking, the partially graphitic nanolayers with broad interlayer distances can provide a reliable pathway for charge carrier transport, which enables fast discharge/charge capabilities [28]. As a result, the lithium storage in the Alg-C electrode may be essentially broken down into a storage in graphene nanolayers (usually below 0.25 V), as well as spectacular storage into turbostratic stacking (above 0.25 V). Unexpectedly, even at a very large current density of 3000 mA g^−1^, the reversible specific capacity of the as-prepared Alg-C anode remains at about 120 mAh g^−1^. This value is much higher than the specific capacities of electrodes containing graphite (~42 mAh g^−1^) and MWCNTs (~25 mAh g^−1^). Because of this, the Alg-C anode has an exceptionally high-rate capability (22.6% retention following a 30-fold increase in current density). Thus, it is believed that the exceptional electrochemical performance of the Alg-C electrode is due to the architectures and intrinsic properties of the unique carbonaceous material.

**Figure 9:**
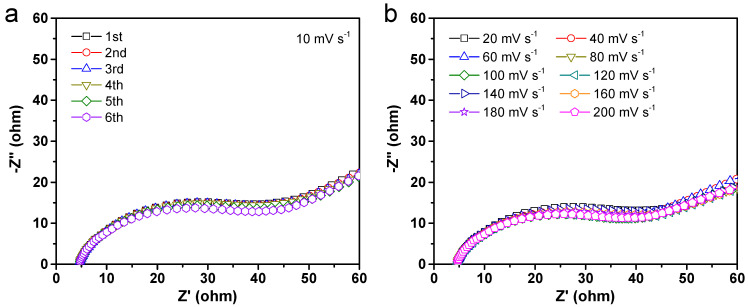
EIS measurement analysis of Alg-C electrode. (**a**) Nyquist plots after different cycles at a scan rate of 10 mV s^−1^. (**b**) Nyquist plots tested at different scan rates ranging from 20 to 200 mV s^−1^.

Because of the remarkable improvement in the intrinsic lithium storage capabilities, we are able to make use of the EIS approach to investigate the transport kinetics of the Alg-C electrode. To be more precise, Nyquist plots were taken after charging in a series of CV scan cycles at different chosen high scan rates from 10 to 200 mV s^−1^. These plots are shown in Figure 9. The resulting Nyquist plots under various test conditions all share similar characteristics. These features are composed of a component that resembles a typical semicircle at high-to-medium frequencies and a component that resembles an inclined line at low frequencies, with form variations that are not particularly noticeable. The indented semicircle depicts the resistance caused by the processes of charge transfer across the electrode interface and lithium diffusion on the surface of the electrode, while the inclined line indicates the resistance caused by lithium diffusion in the electrode itself [8,57]. Briefly, the surface resistance steadily rises as the number of continuously operating CV scan cycles accumulates (Figure 9a). However, the charge transfer resistance undergoes a slight decrease after each cycle of discharging/charging the battery, particularly after cycling at a high CV scan rate (Figure 9b). The electrochemical impedance hardly changes at all, even after being scanned at a rate of up to 200 mV s^−1^, which indicates that the SEI layer does not significantly expand even when subjected to severe circumstances [57]. Based on this point of the previous discussions, it is possible to conclude that the cycled Alg-C electrode has a reduced electrochemical resistance. This is consistent with the outcomes of galvanostatic discharge/charge tests and may improve the electrode response kinetics during repeated lithiation/delithiation operations at high current density.

**Figure 10:**
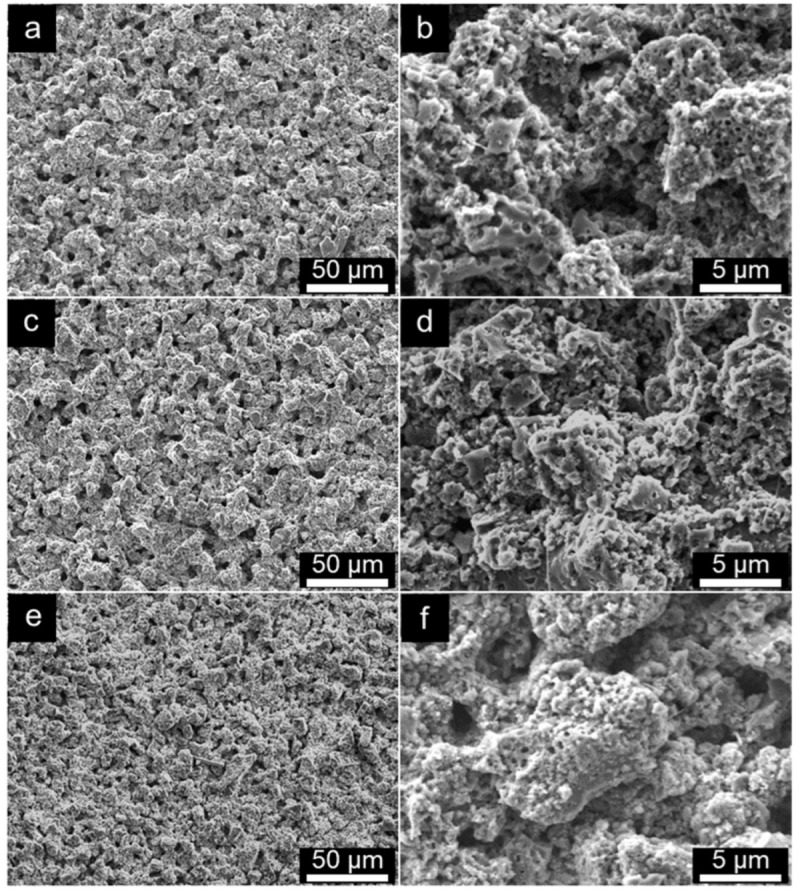
SEM images of Alg-C electrodes. (**a**,**b**) Fresh electrode; (**c**,**d**) after the 100th cycle; (**e**,**f**) after the 5000th cycle at a current density of 500 mA g^−1^ between the potential window from 0.02 to 3.0 V.

**Figure 11:**
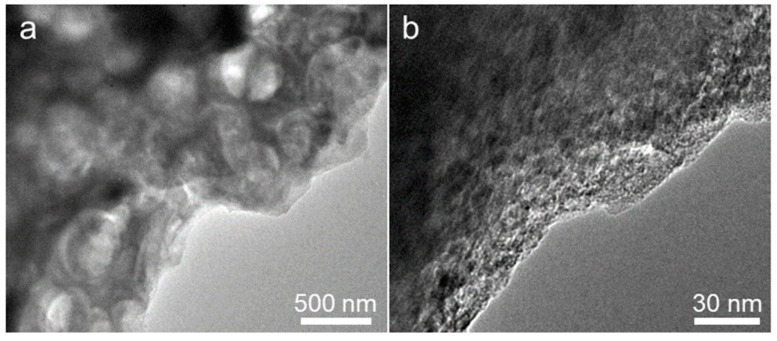
(**a**) Low and (**b**) high magnifications TEM images of Alg-C electrode after the 5000th cycle at a current density of 500 mA g^−1^.

The SEM technique was used to further explore the morphological and structural evolution of the Alg-C anode while it was subjected to extreme volume expansion and contraction during lithium insertion and extraction. Figure 10 illustrates the SEM morphologies of the three Alg-C electrodes before cycling, and after the 100th and 5000th cycles at a current density of 500 mA g^−1^ shown in Figure 7b. These images may provide further objective information on the microscale effects of electrochemical processes. The as-prepared Alg-C anode still maintains its integrity and is comparable to that of the pristine anode even after extremely long deep discharge/charge cycles, as demonstrated by the SEM images of the fresh electrode (Figure 10a,b), after the 100th cycle (Figure 10c,d), and after the 5000th cycle (Figure 10g,h). This discovery makes it abundantly evident that the structural maintenance of the Alg-C electrode significantly contributes, among other factors, to the increase in capacity retention during electrochemical discharge/charge processes at voltages ranging from 0.02 to 3.0 V. In addition, the TEM images illustrated in Figure 11 demonstrate that the hierarchically porous structure does not change much even after being subjected to 5000 cycles of discharge/charge, indicating that it retains the same characteristics as it has before cycling. In addition, additional investigation indicates that there has been a small change in the structure of Alg-C, which is consistent with what one would anticipate after an extensive period of testing that included multiple cycles. Because their shapes are comparable, it is believed that the Alg-C anode has the ability to significantly reduce the amount of considerable cracking that occurs during cycling. This ensures that their electrochemical performance is exceptional. Moreover, surface functional groups cannot be ignored when evaluating the electrochemical characteristics of a material since they influence the wettability of both the electrolyte and the material surface.

The improved lithium storage capability of the as-prepared Alg-C may have been primarily attributable to the following beneficial characteristics of the material. (1) The bio-derived nanostructured porous carbon has a large surface area, which could provide easy access to electrolytes with multiple active sites, enhancing ionic/electronic transport properties. This is because of the increased surface area of the active sites. (2) The unique amorphous carbon matrices that include partly graphitic nanolayers could accommodate an increased number of lithium storage sites, such as nanopores and cavities. This would result in a greater capacity. (3) The incorporated oxygen-containing functional groups on the Alg-C sample are able to make unquestionable contributions to play positive roles in subsequent faradaic reactions. This is because they are able to incorporate oxygen into their structure. Additionally, theoretical simulations and advanced in situ characterization techniques are needed to investigate the lithium storage mechanism and to gain insight into the relationship between the bio-derived Alg-C structure and performance to better optimize the design of electrode materials. Considering that more than 20000 tons of Na-alginate can be extracted from seaweed for industrial use each year and that carbon-related materials can be produced at low cost using environmentally friendly conditions, this fabrication process could easily be extended to the battery materials industry [58,59].

## 4. Conclusions

In summary, oxygen-doped hierarchically porous carbon with partially graphitic nanolayers could be effectively generated by pyrolyzing a natural Na-alginate biopolymer without the assistance of any extra activation step. When applied as an anode material in LIBs, the Alg-C electrode exhibits high specific capacity, superior rate capability (120 mAh g^−1^ at 3000 mA g^−1^), and excellent cycling stability over 5000 cycles. The excellent performance of Alg-C may be attributed to its novel structural characteristics as well as the abundance of oxygen functional groups. Additional research is required to determine the capacity losses that occur during the initial few cycles and develop strategies to reduce their impact. Therefore, these findings highlight the potential of bio-derived Alg-C materials as interesting anodes suitable for high-performance rechargeable LIBs.

## Data Availability

The data presented in this study are available on request from the corresponding author.
